# GS-Chaff: Multi-Agent Prompt-Level Semantic Chaffing for Privacy-Preserving LLM Inference

**DOI:** 10.3390/s26175385

**Published:** 2026-08-26

**Authors:** Quan Zhou, Zhicheng Wang, Zhe Yue, Libin Cai, Kuien Liu, Caiyan Qin

**Affiliations:** 1 School of Mathematical Sciences, Sichuan Normal University, Chengdu 610068, China; quan28186@gmail.com; 2 School of Computer Science and Engineering, University of Electronic Science and Technology of China, Chengdu 611731, China; 202552080537@std.uestc.edu.cn (Z.W.); yuezii01@gmail.com (Z.Y.); 2024091202004@std.uestc.edu.cn (L.C.); 3 Institute of Software, Chinese Academy of Sciences, Beijing 100190, China; kuien@iscas.ac.cn; 4 School of Robotics and Advanced Manufacture, Harbin Institute of Technology, Shenzhen 518055, China

**Keywords:** multi-agent, generative semantic chaffing, prompt obfuscation, small language model, large language model

## Abstract

Cloud-based large language model (LLM) services are increasingly used to process natural-language queries that may contain private or sensitive information. Conventional privacy-preserving approaches, such as cryptographic protection and text sanitization, often introduce substantial computational overhead or disrupt the semantic integrity of the original query, resulting in a trade-off between privacy protection and task utility. To address this limitation, we propose generative semantic chaffing (GS-Chaff), a training-free multi-agent framework for privacy-preserving LLM inference over natural-language text queries. Rather than explicitly masking sensitive content, GS-Chaff hides the user’s true intent among semantically plausible chaff queries. The framework is implemented through two small language model (SLM)-based agents: a privacy policy agent that adaptively determines the required semantic abstraction level and chaffing factor for each text query, and a generative semantic chaffing agent that produces semantically aligned dummy queries. After cloud-side inference, the response corresponding to the protected real query is recovered locally using a stateless index, without modifying the cloud-based LLM. Experimental results on text-based benchmarks demonstrate that GS-Chaff reduces the attacker’s real-query identification rate to 22.5%, close to random guessing, while maintaining inference utility on the evaluated benchmarks. In addition, GS-Chaff reduces local preprocessing time by 1.85× compared with a fixed chaffing configuration using β=5.

## 1. Introduction

With the rapid advancements in scalable language modeling, cloud-based LLM services offer an increasing number of utilities for human assistance [1,2]. The interactivity offered by these models allows a high degree of freedom in human–AI interaction, permitting users to share diverse forms of information with the model, including private and sensitive data [3,4]. For high-stakes information, such as legal, healthcare, and financial content, this level of utility may pose serious risks to privacy and data protection [5]. Sensitive data transmitted as semantic queries during inference can potentially be extracted by adversaries through model inversion, query exploitation, or server-side data leakage, making it vulnerable to misuse [6]. Moreover, the obscure data handling practices of cloud-based services limit users’ ability to verify how their information is stored, processed, or retained over time [7,8]. Together, these concerns expose a fundamental trade-off between usability and privacy in cloud-assisted semantic inference systems.

Studies in privacy-preserving computation have developed over the last few decades. Conventional methods are dominated by cryptographic mechanisms, such as homomorphic encryption (HE) and secure multi-party computation (MPC) [9,10]. Despite being theoretically strong, these methods involve a high computation cost that makes them infeasible for scalable implementation on LLMs [11]. In resource-constrained edge environments, the substantial computational and communication overhead of heavyweight cryptographic protocols may limit their applicability to latency-sensitive LLM inference [12]. Besides the cryptographic method, text sanitization is often implemented in contemporary privacy-preserving attempts [13]. This method identifies sensitive entities from the user prompt and replaces those with a non-sensitive term with similar semantic meaning. Despite its simplicity and effectiveness, text sanitization causes the obfuscated prompt to lose its true contextual meaning. This harms the LLM’s understanding and thus forces the model to produce a generic answer instead of a tailored one for a user’s specific case. Moreover, this simple replacement strategy is highly vulnerable to re-identification attacks based on background knowledge [14].

Protecting user privacy at the semantic level therefore requires regulating how intent is represented and conveyed during interaction with cloud intelligence services [15,16]. LLM-based multi-agent systems enable the decomposition of complex reasoning into specialized components, allowing agents to collaborate and perform diverse tasks in parallel [17]. This modular structure supports flexible adaptation to different contexts and fine-grained control over processing steps, which reduces potential information leakage. Recent work applies this approach to privacy-sensitive tasks, including semantic-level query obfuscation [18] and encrypted inference configuration [19], showing that collaborative agents protect sensitive information without substantially degrading task performance.

Motivated by this observation, we propose a privacy protection method that shifts from content hiding to intent hiding. Inspired by Rivest’s classic “Chaffing and Winnowing” theory [20], we conjecture that in the absence of strong encryption, the best way to protect privacy is to obfuscate real sensitive query with the presence of noisy yet semantically plausible queries. However, migrating this bit-level theory to semantic-level applications involving communication-mediated query representations faces significant challenges. Particularly, attackers can easily eliminate generated forged queries through statistical analysis if these queries are not natural enough in terms of syntax, logic, or domain distribution.

**Figure 1:**
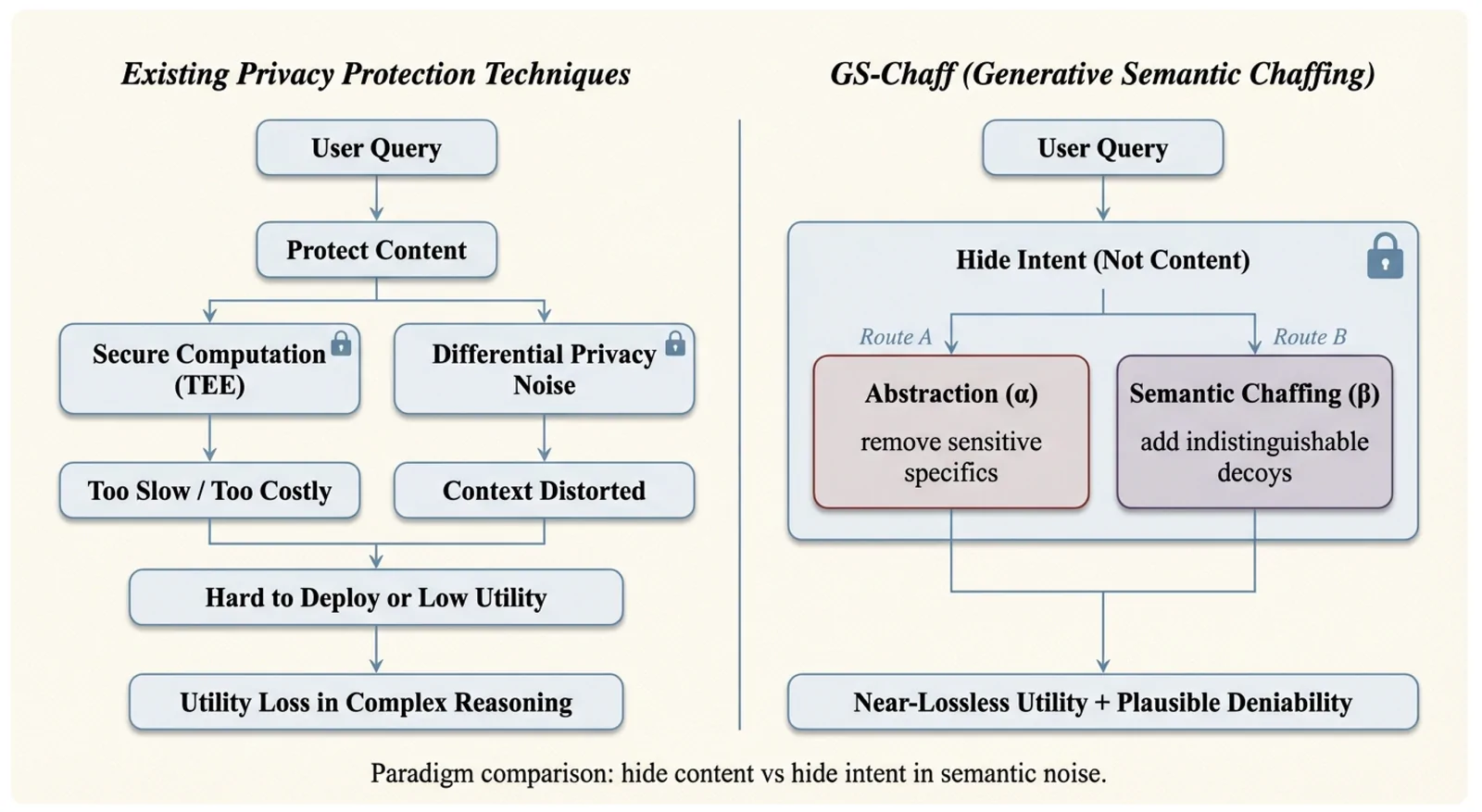
Comparison between our work and previous studies in privacy-preserving LLM inference. GS-Chaff addresses the challenges of computation complexity in secure computation method and broken semantic context in differential privacy method. Specifically, we protect the users’ confidentiality in the prompt by hiding their true intents among the forged queries.

Considering these challenges, we propose generative semantic chaffing (GS-Chaff), a training-free privacy-preserving inference framework implemented as a lightweight SLM-based multi-agent system inspired by chaffing and winnowing theory. Instead of performing black-box encryption on cloud LLMs, GS-Chaff deploys two cooperative SLM-based agents to construct a semantic-space defense layer over heterogeneous textual query realizations during transmission, leveraging their instruction-following capability and computational efficiency. The use of SLMs enables efficient client-side processing of abstraction-aware text queries prior to cloud inference while keeping the preprocessing pipeline lightweight. Specifically, GS-Chaff consists of a privacy policy agent and a generative semantic chaffing agent. The privacy policy agent dynamically determines an appropriate level of abstraction (α) and chaffing factor (β) according to query sensitivity and task requirements, seeking a balance among semantic abstraction, query obfuscation, and inference utility. The generative semantic chaffing agent produces semantically plausible chaff queries that align with the protected query in granularity and stylistic distribution. Through coordinated agent collaboration, GS-Chaff enables the cloud LLM to perform blind inference over mixed textual queries, making it difficult for adversaries to statistically identify the user’s true intent. Figure 1 illustrates the conceptual comparison between GS-Chaff and existing approaches to privacy-preserving LLM inference.

We summarize our contributions as follows:

- We are among the first to formulate privacy-preserving LLM inference as an intent-hiding problem in text-query transmission and to implement this idea through an SLM-based multi-agent architecture rather than explicit content masking.
- We propose GS-Chaff, an efficient and training-free privacy protection mechanism inspired by chaffing and winnowing theory, which balances privacy and utility by concealing user intent through the generation of semantically plausible textual chaff queries.
- Through extensive experiments on text-based benchmarks, we demonstrate that GS-Chaff reduces the adversarial identification rate to 22.5%, close to the random-guessing level, while maintaining task utility on the benchmarks. In addition, GS-Chaff achieves a 1.85× reduction in local preprocessing time compared to the fixed β=5 chaffing configuration.

## 2. Related Work

***Privacy-Preserving Inference on LLMs.***  Privacy-preserving inference aims to enable LLM services without revealing user inputs or intermediate reasoning traces to the service provider or external adversaries [21]. Early foundations in this area draw from secure multi-party computation (MPC), which enables joint computation over private inputs without disclosure [22], and fully homomorphic encryption (FHE), which allows arbitrary computation directly on encrypted data [23]. To address statistical leakage, differential privacy (DP) has been explored as a mechanism to limit information exposure during model interaction and training [24]. More recently, these cryptographic and statistical principles have been adapted to neural network inference and LLMs, enabling encrypted transformer execution and confidential prompting in cloud settings [25,26]. In sensor communication privacy, PrivacyOracle leverages large language models to configure privacy firewalls for pervasive sensing infrastructures, enabling automated reasoning over contextual norms and filtering sensitive information flows in smart built environments [27].
***Cryptographic Protection.***  Cryptographic approaches have recently been explored to enable privacy-preserving LLM inference in distributed or cloud-based deployment settings. FLUTE [26] proposes a secure two-party inference framework that partially encrypts transformer components to reduce communication and computation overhead. Socratic CoT [28] combines homomorphic encryption with encrypted retrieval to protect both user queries and intermediate reasoning traces. Power-Softmax [29] introduces a cryptographically friendly reformulation of the softmax operation to improve the efficiency of secure inference under encryption. TFHE-Coder [30] studies the feasibility of LLM agents to automatically generate FHE programs, highlighting the challenges of correctness and performance in encrypted computation. PipeLLM [31] proposes speculative encryption to overlap communication and computation, significantly improving throughput for confidential LLM services. FHE-Agent [19] leverages an LLM-guided agentic framework to automate CKKS parameter configuration, reducing the deployment complexity of encrypted inference pipelines. Despite their strong privacy guarantees, these cryptographic approaches generally incur substantial computational overhead, require specialized hardware or careful parameter tuning, and remain difficult to scale to interactive, low-latency, and reasoning-intensive LLM applications.
***Text Sanitization.***  Text sanitization has emerged as a lightweight alternative for privacy-preserving LLM inference by modifying user inputs to remove or obfuscate sensitive information before model execution. DYNTEXT [32] proposes a semantic-aware dynamic sanitization framework that adapts redaction strategies based on contextual sensitivity to balance privacy and utility. ZSTS [33] explores zero-shot redaction and substitution using LLMs, demonstrating improved generalization across domains without task-specific tuning. Self-Sanitize [34] shifts the sanitization focus from inputs to model outputs, mitigating privacy leakage during response generation. Another work [35] demonstrates the feasibility of applying LLM-driven sanitization in sensitive healthcare settings. While text sanitization methods are efficient and easy to deploy, they often disrupt semantic coherence, degrade task-specific reasoning, and remain vulnerable to re-identification and contextual reconstruction attacks, limiting their reliability for high-stakes LLM inference [36]. These limitations are also observed in other studies that explore the utility degradation introduced by sanitization under limited computational budgets [37], and how word-level differential privacy sanitization can introduce contextual vulnerabilities that enable partial reconstruction attacks [38].
***Prompt Obfuscation.***  Prompt obfuscation has been explored as a pragmatic privacy protection strategy that conceals sensitive user intent while preserving LLM usability. EmojiPrompt [39] introduces a generative prompt obfuscation framework that encodes sensitive content into emoji-based representations, enabling privacy-preserving communication with cloud-based LLMs. Petridish [40] proposes an obfuscation–de-obfuscation pipeline that transforms user prompts into semantically equivalent but unintelligible representations before cloud inference. Lock and Decode [41] frames prompt obfuscation as a cryptographic-like mechanism, demonstrating how obfuscated prompts can bypass or reshape LLM security controls. WordGame [42] studies simultaneous obfuscation of both queries and responses, revealing how such transformations can evade LLM safeguards while maintaining functional interaction. CodeCipher [43] learns task-aware code obfuscation strategies to prevent LLMs from extracting sensitive implementation details from source code. Despite their flexibility and low deployment cost, prompt obfuscation methods typically lack formal privacy guarantees, are vulnerable to semantic inversion and adaptive attacks, and often introduce instability or performance degradation in reasoning-intensive LLM tasks. Table 1 summarizes these representative prompt-obfuscation methods.

Unlike these approaches, which primarily transform, encode, or obfuscate an individual prompt, GS-Chaff preserves natural-language interaction and conceals the protected query within a dynamically generated batch of semantically plausible alternatives through query-adaptive semantic abstraction and chaff generation.

***AI Agent-based Privacy Protection***  Multi-agent systems powered by LLMs have recently been explored as a mechanism to actively preserve privacy during inference. The GAMA framework proposes a general anonymizing multi-agent architecture that separates private and public processing spaces, using domain rules and logic enhancement to protect sensitive information while maintaining task performance [44]. SecureGov-Agent introduces a governance-centric framework designed to mitigate privacy leakage and adversarial attacks through centralized monitoring, risk scoring, and content analysis, achieving reduced privacy exposure with moderate impact on task completion [45]. PrivAct internalizes contextual privacy preferences into individual agent policies, demonstrating improved privacy-utility trade-offs across diverse multi-agent topologies by embedding privacy constraints into agents’ generation behavior [18]. In addition, architectural frameworks such as Sentinel Agents incorporate distributed security layers that continually analyze semantic behavior and cross-agent communication to detect and contain privacy breaches and other threats [46]. These approaches highlight the potential of embedding privacy control within adaptive, communication-aware inference pipelines.

## 3. Method

### 3.1. Problem Definition

We consider confidential LLM inference as a safe interaction between a user and a cloud-based server. On the local side, sensitive queries are initiated through SLM-driven agents that mediate private interaction. This query is passed to the powerful LLM in the server side to obtain high-quality inference results while maximizing privacy protection at low computational cost. The LLM at the server side naturally will record all input information, which includes user’s true intent, identity, and sensitive attributes. This typical behavior makes the interaction prone to threat from attackers, which we assume to include the following:

- re-identification attack based on semantic distribution, where the attacker attempts to identify a real user’s intent by analyzing semantic fine-grainedness, entity distribution, and contextual consistency of the queries;
- discriminative attack based on the consistency of the language model, where the attacker evaluates the naturalness, realness, and rationality of the queries by leveraging language models;
- multi-agent consensus attack, where the attacker deploys multiple LLM agents to perform parallel analysis on the same query batch and improve the discrimination confidence level through voting.

### 3.2. Threat Model

We consider an honest-but-curious cloud LLM service provider as the primary adversary. The provider follows the inference protocol and returns responses for all submitted queries. The provider observes the complete plaintext query batch, the shuffled order, and the corresponding responses. The provider knows the GS-Chaff procedure, the local SLM configuration, the prompt templates, and the ranges of α and β. Available attack tools include language models, semantic features, classifiers, and multiple cooperating agents. The provider has no access to the locally stored real-query index or other client-side state that is not transmitted to the cloud.

GS-Chaff does not conceal query plaintext from the cloud provider because the cloud LLM processes every query in the batch. The protected asset is the association between the user and the query representing the user’s actual intent. The privacy objective is therefore real-query identification resistance, intent hiding, and plausible deniability rather than complete query confidentiality or user anonymity.

The threat model distinguishes adversary identities, attack strategies, and attack scopes. An external observer observes communication metadata, while encrypted transport prevents access to plaintext queries. An adaptive semantic classifier trains a dedicated classifier or ranker using labeled GS-Chaff samples. A multi-agent attacker aggregates the predictions of multiple LLM agents analyzing the same shuffled query batch. A side-channel attacker uses non-semantic features to infer the real query position. A cross-session profiler correlates multiple query batches using identity, session, historical, entity, and temporal information. Our experiments focus on single-batch multi-agent identification and side-channel classification using token length and end-to-end request latency.

The experiments focus on single-batch real-query identification under the multi-agent and limited side-channel settings described above. Across these settings, the attack objective and success criterion are defined consistently. Given the *i*-th shuffled query batch containing $\beta_{i}$ queries, the attacker outputs a predicted real-query index ${\hat{k}}_{i}$. An attack succeeds when ${\hat{k}}_{i}$ equals the locally stored real-query index $k_{i}^{*}$. We use Top-1 Real-Query Identification Accuracy as the primary attack-oriented privacy metric. A lower identification accuracy indicates stronger privacy protection. We additionally report the Privacy Protection Rate (PPR), defined as

(1) $$\mathrm{PPR}=1-{\mathrm{Acc}}_{\mathrm{ID}},$$

where ${\mathrm{Acc}}_{\mathrm{ID}}$ denotes the Top-1 Real-Query Identification Accuracy. Accordingly, a higher PPR indicates stronger privacy protection. When reported as percentages, the corresponding relation is $\mathrm{PPR}\left(\%\right)=100-{\mathrm{Acc}}_{\mathrm{ID}}\left(\%\right)$.

GS-Chaff assigns a sample-specific chaffing factor $\beta_{i}$. Therefore, the random-guessing baseline for Top-1 Real-Query Identification Accuracy over *N* samples is calculated as

(2) $${\mathrm{Acc}}_{\mathrm{ID}}^{\mathrm{random}}=\frac{1}{N}\sum_{i=1}^{N}\frac{1}{\beta_{i}}.$$

### 3.3. Technical Framework

**Figure 2:**
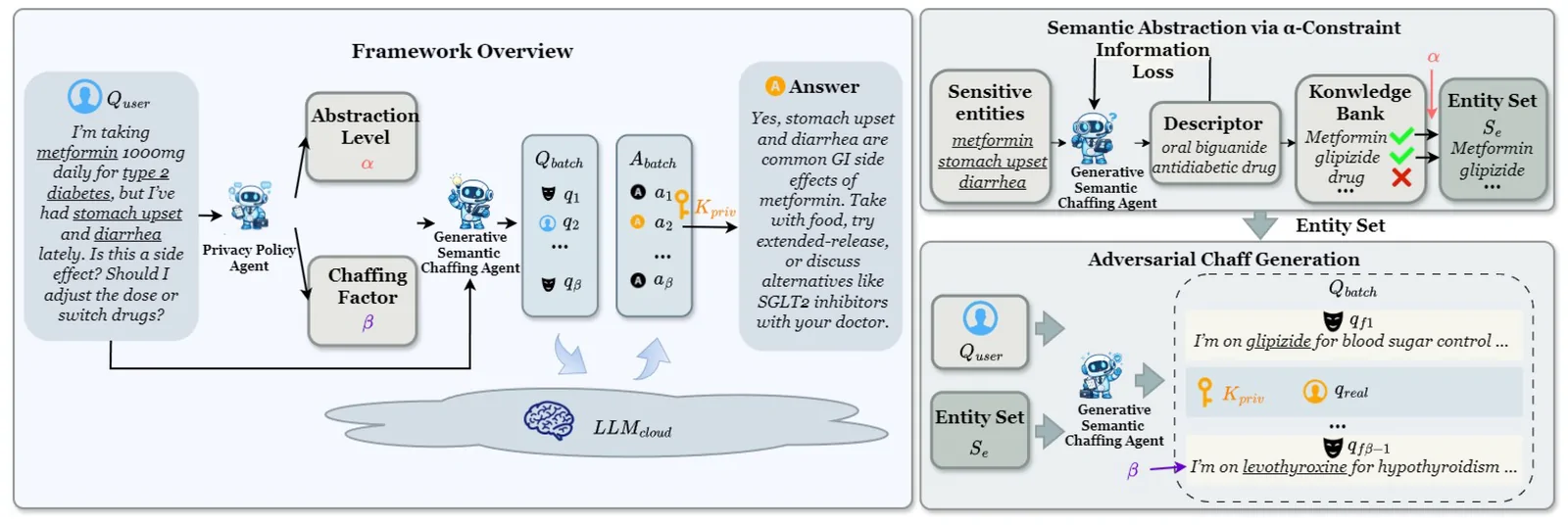
Framework of GS-Chaff that leverages hiding user intent as its main objective. GS-Chaff obfuscates real query with two modules, privacy policy controller that determines the level of privacy, and generative forger that generates noisy queries. We leverage SLM to achieve optimal privacy protection at minimum inference cost.

To address the above threat challenges, we propose GS-Chaff, a lightweight multi-agent framework that provides privacy protection for natural-language text queries transmitted to cloud-based LLMs. Inspired by Rivest’s “screening and chaffing” theory [20], GS-Chaff extends conventional bit-level chaffing to the semantic space. The framework is composed of three stages: adaptive privacy policy, generative semantic chaffing, and oblivious inference with winnowing. Figure 2 illustrates the overall design.

#### 3.3.1. Privacy Policy Agent

Conventional privacy protection methods generally adopt static protection strategies that may lead to a privacy–utility imbalance. In contrast, we apply a dynamic protection strategy by leveraging an SLM-based policy agent driven by specialized prompts to determine query-specific privacy parameters. The policy agent selects these parameters through instruction-guided inference.

Specifically, given an input query *q*, the SLM outputs a decision vector θ as follows:

(3) θ=〈α,β〉,

where α,β∈{1,…,10}. The parameters α and β define the dynamic privacy policy as follows:

- α refers to the abstraction level that must be covered by the descriptor. A larger α corresponds to a more generalized semantic description, thereby reducing reliance on fine-grained information.
- β refers to the chaffing factor, representing the total number of queries sent to the server. These queries consist of one protected real query and β−1 chaff queries. Increasing β increases the uncertainty of the real query within the transmitted batch, while introducing additional computational and communication overhead.

We apply the following prompt to the SLM to obtain the decision vector.

The policy agent directly selects α and β through instruction-guided SLM inference according to query sensitivity, domain characteristics, and reasoning requirements. The parameters are not obtained through explicit numerical optimization, exhaustive search, or random sampling. The resulting query-adaptive policy provides an empirical trade-off among privacy protection, task utility, and inference overhead.

The policy output is parsed as a single parameter pair 〈α,β〉. A valid output must contain two integers within the range [1,10]. Invalid, incomplete, or out-of-range outputs trigger a format-correction retry. If the regenerated output remains invalid, the predefined fallback policy 〈3,5〉 is used. This instruction-guided decision process is implemented using the prompt template shown in Listing 1, while the complete policy selection and validation procedure is summarized in Algorithm 1.
**Algorithm 1** Privacy Policy Selection**Require:** Query *q* **Ensure:** Policy parameters 〈α,β〉
  1:y←SLM(q)  2:〈α,β〉←Parse(y)  3:**if** α∉{1,…,10}∨β∉{1,…,10} **then**  4:      Regenerate *y* using a format-correction instruction  5:      〈α,β〉←Parse(y)  6:**end if**  7:**if** α∉{1,…,10}∨β∉{1,…,10} **then**  8:      〈α,β〉←〈3,5〉  9:**end if**10:**return** 〈α,β〉

**Listing 1.** Prompt template used by the privacy policy agent
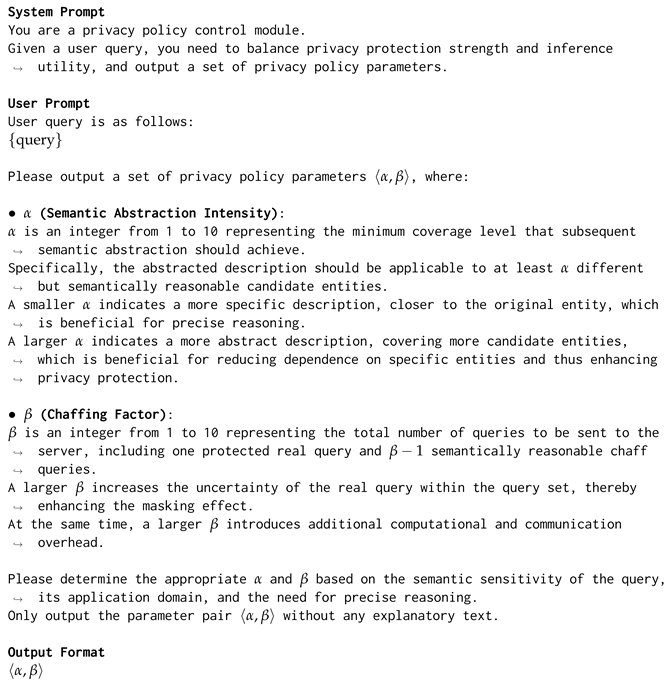


#### 3.3.2. Generative Semantic Chaffing Agent

We leverage another SLM as a generative semantic chaffing agent, driven by dedicated prompts, to translate the decision vector θ from the policy agent into semantic payloads. The agent produces semantically plausible noisy queries guided by the privacy policy agent’s 〈α,β〉. Each textual query is represented as a high-dimensional semantic embedding, capturing its abstract semantic content. The generated noisy queries occupy the same semantic space and act as alternative textual realizations, making the real query difficult to distinguish from the other queries in the transmitted batch. Specifically, SLM performs semantic abstraction and chaff generation.

***Semantic Abstraction.*** To prevent the server from inferring user identities through specific entities, the SLM performs semantic abstraction on sensitive entity *e* in the query *q*. Specifically, given the abstraction parameter α determined by the privacy policy agent, the SLM generates a descriptor *D* that generalizes the sensitive entity while preserving task-relevant semantic information. The parameter α serves as a prompt-level semantic abstraction target: a larger α instructs the SLM to generate a broader descriptor compatible with more semantically reasonable candidate entities, whereas a smaller α retains more fine-grained information about the original entity. The abstraction is performed through prompt-guided SLM generation rather than explicit enumeration over a knowledge base or formal entailment and semantic-distance verification.
***Adversarial Generation.*** To further conceal underlying user intention, especially when α is small, SLM generates β−1 adversarial chaff queries. We are motivated by semantic granularity alignment, where, according to encryption theory, attackers can easily eliminate fake data if the distribution of fake data does not match that of real data. Thus, fake query $q_{f}$ must strictly adhere to the same semantic constraints as the abstracted real query $q_{r}^{\prime }$. SLM generates a set of fake queries ${\left\{q_{i}^{f}\right\}}_{i=1}^{\beta -1}\in Q_{f}$, which must satisfy the following constraints:
  - plausibility, $P_{LM}\left(q_{f}\right)\approx P_{LM}\left(q_{r}^{\prime }\right)$, where the probability distribution of the language model is similar for both real and fake queries,
  - fake entity $e^{\prime }$ in the fake query $q_{f}$ must also be a descriptor that has undergone α-abstraction.

Therefore, we formalize the chaff generation as follows.

(4) $$\begin{array}{c} q_{f}∼SLM\left(\cdot |T(q_{r}^{\prime }),S=\mathrm{Med},C=\alpha \right) \end{array}$$

where T, S, and C refer to template, style, and constraint. This mechanism aims to reduce differences among the β transmitted queries in semantic granularity, terminology, and stylistic form, rather than providing a formal guarantee that all queries belong to an identical embedding cluster.

#### 3.3.3. Oblivious Inference and Winnowing

This module performs parallel server-based inference and local extraction of the real result using a locally retained selection index. This mechanism is conducted through two stages: local selection index generation and shuffling, and server execution with local winnowing. Unlike Rivest’s original MAC-based chaffing-and-winnowing construction, GS-Chaff uses the locally retained real-query index only for response selection and does not provide MAC-based authentication or integrity guarantees.

***Local Selection Index and Shuffling.*** Before transferring to the server, the local system performs an obfuscation operation. Specifically, we perform a random permutation on the query set consisting of the abstracted real query $q_{r}^{\prime }$ and the set of fake queries $Q_{f}$:
  (5) $$Q_{\mathrm{batch}}={\mathrm{Permute}}_{\pi }\left(\left\{q_{r}^{\prime }\right\}\cup Q_{f}\right).$$

The local selection index is defined as the position of the protected real query in the shuffled batch:

(6) $$K_{\mathrm{sel}}={\mathrm{Id}}_{\pi }\left(q_{r}^{\prime }\right).$$

Only this small integer index needs to be retained locally for subsequent response selection, without maintaining additional cryptographic state.

***Server Execution and Local Winnowing.*** The server-side model $M_{\mathrm{server}}$ performs inference over all queries in the shuffled batch, producing
  (7) $$A_{\mathrm{batch}}=\left\{M_{\mathrm{server}}\left(q\right)|q\in Q_{\mathrm{batch}}\right\}.$$

After receiving $A_{\mathrm{batch}}$, the client uses the locally retained selection index $K_{\mathrm{sel}}$ to extract the response corresponding to the protected real query:

(8) $$a_{\mathrm{final}}=A_{\mathrm{batch}}\left[K_{\mathrm{sel}}\right].$$

### 3.4. Theoretical Analysis

***Plausible Deniability.*** Under an idealized setting in which the protected query and the β−1 chaff queries are indistinguishable to the attacker and have equal prior probability, the probability of identifying the protected query by random selection is
  (9) $$P\left(\mathrm{Real}=q_{i}|Q_{\mathrm{batch}}\right)=\frac{1}{\beta }.$$

In practice, the protected and chaff queries are not guaranteed to be exchangeable or identically distributed, and lexical, semantic, or contextual differences may provide additional information to an attacker. Therefore, 1/β is used as an idealized random-guessing reference rather than a formal posterior bound, and the actual identification risk is evaluated empirically.

***Utility Considerations.*** Unlike conventional naïve replacement methods, GS-Chaff uses the abstraction parameter α to control the degree of semantic generalization. A smaller α preserves more query-specific information and is therefore expected to incur less utility degradation, whereas a larger α provides stronger abstraction at a potential utility cost. This relationship is treated as a design intuition rather than a formal utility guarantee and is evaluated empirically in our experiments.

## 4. Results

This section elaborates on the evaluation of our GS-Chaff method. To test the effectiveness of our method, we leverage Llama-3.2-1B as the SLM. We utilize a diverse set of LLMs to perform inference, ranging from open-sourced to proprietary models as follows:

- Local inference: Llama2-7B, Vicuna-13B
- Cloud inference: GPT-3.5-Turbo, GPT-4-Turbo, and GPT-4o

Note that we set the temperature of policy SLM to zero to ensure the stability of output results under the same input. For experiments reported over three independent runs, we use random seeds 2024, 2025, and 2026. For proprietary cloud APIs, decoding parameters not explicitly specified in the corresponding experiments are left at the provider-default settings. Baseline methods are implemented following the methodological descriptions in their corresponding references; when official implementations or complete configurations are unavailable, we follow the descriptions provided in the cited works and evaluate them under the same benchmark and cloud-model settings.

### 4.1. Main Results

We compare our method against several strong baselines, such as no privacy protection, local differential privacy (i.e., Text2Text [47] and Paraphraser [48]), and ConfusionPrompt [49]. We evaluate GS-Chaff on both general reasoning (i.e., StrategyQA [50] and MuSiQue [51]) and domain-specific benchmarks (i.e., MedQA [52] and LegalBench [53]). We utilize the following metrics for evaluation: accuracy for StrategyQA, MedQA, and LegalBench; F1, ROUGE-L, and exact match (EM) for MuSiQue; and AUC for StrategyQA. Table 2 demonstrates that GS-Chaff consistently outperforms other privacy-preserving methods across all cloud models, achieving utility scores nearly identical to the non-private baselines (e.g., 0.865 on MedQA with GPT-4o).

***Adversarial Detection.*** We evaluate query identification under a multi-agent consensus attack. Two GPT-4-Turbo attacker agents receive the same shuffled query batch and independently predict the index of the query that represents the user’s actual intent. The attacker agents know that the batch contains one real query and multiple GS-Chaff-generated queries. Their predictions and explanations are submitted to an arbitration agent, which produces the final predicted index. When the attacker agents disagree, the arbitration agent determines the final prediction based on their analyses. An attack succeeds when the final predicted index equals the locally stored real-query index. We report the real-query identification rate over three independent runs on LegalBench. Table 3 shows that GS-Chaff achieves a real-query identification rate of 22.5 ± 1.8%, which is close to the empirical random guess baseline (21.2 ± 1.5%), indicating strong resistance under the evaluated multi-agent consensus attack setting across multiple trials.

### 4.2. Ablation Studies

***Component Contribution.*** We conduct ablation to measure the effectiveness of components in our framework. For this experiment, we evaluate the following conditions, static protection with α=3 and β=5 (static), static α, static β, replacing α-guided semantic abstraction with random token masking (w/o α), and removing chaff queries (w/o β).

We measure answer accuracy (Acc.) and Privacy Protection Rate (PPR), where a higher PPR indicates stronger privacy protection. Table 4 shows that setting the value dynamically for both α and β is essential for our framework to yield the best performance on privacy protection. Further, we also demonstrate that GS-Chaff maintains consistent performance across the two SLM backbones included in this ablation, Qwen2.5-1.5B and Llama-3.2-1B.

**Figure 3:**
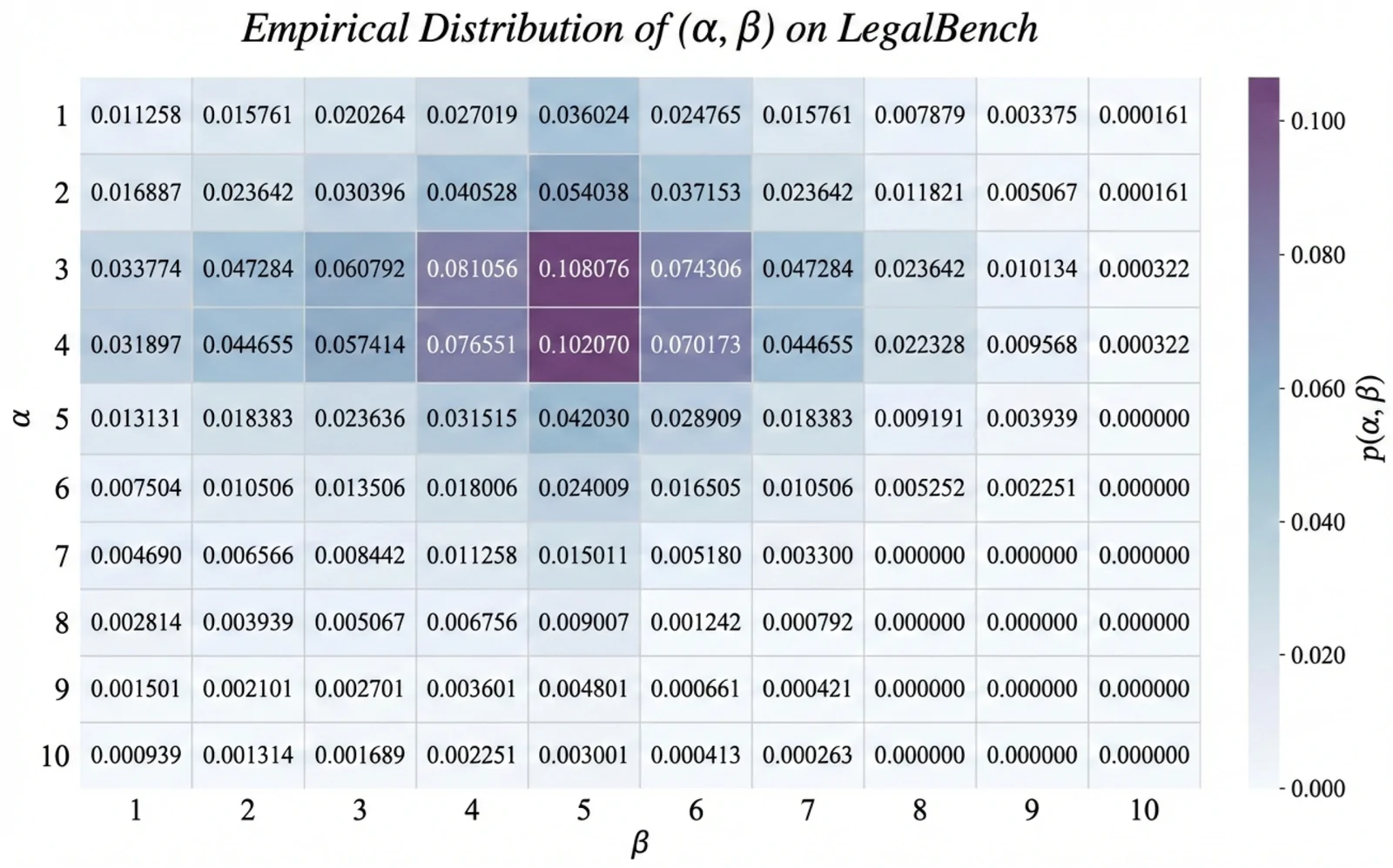
Joint distribution of privacy policy output.

***Dynamics of Policy.*** To evaluate the behavior of dynamic privacy policy, we calculate the empirical joint distribution of 〈α,β〉 output by the policy SLM for samples on the LegalBench dataset. Figure 3 presents the joint distribution of privacy policy $p\left(\alpha ,\beta \right)$, normalized, with the sum of all cells equal to 1. We observe that the probability mass of α is mainly concentrated in the range of 3–4, while β appears most frequently around 5. This indicates that the policy agent tends to select moderate semantic abstraction levels to balance privacy protection and reasoning utility. Meanwhile, a relatively higher β is chosen to introduce sufficient chaff queries, increasing uncertainty about the real user intent without imposing excessive computational overhead. Furthermore, the combination of high α and β hardly ever occurs. The results illustrate the empirical trade-off between abstraction strength and obfuscation strength, supporting the use of a dynamic policy rather than a fixed parameter configuration. This self-adaptive behavior is consistent with the adversarial results approaching random guessing in Table 3.
***Side-Channel Attack.*** We evaluate a limited side-channel attacker that uses only per-query token length, end-to-end request latency, or their combination to predict the real-query position. To verify the effectiveness of our intent-hiding objective for privacy-preserving inference, we perform experiment on side-channel attack. We measure the success rate of real query identification in query batch through side-channel signals. Specifically, the attacker observes per-query token length and end-to-end request latency to predict the index of real query. We perform the experiment on LegalBench with 500 samples. For each query, GS-Chaff generates queries with a batch size of β=5. We train a lightweight classifier to predict the index of real query in the batch. We report Top-1 identification accuracy (Top-1 acc.) and mean reciprocal rank (MRR). Table 5 shows that combined side-channel attack, with both token length and latency signals, achieve identification accuracy only marginally better (3.5-point) than random guess. The result indicates that GS-Chaff does not rely on hiding superficial properties such as query length and execution time. Its effectiveness stems from semantic-level intent obfuscation.

### 4.3. Additional Evaluation

***Human Evaluation.*** We perform a manual evaluation on 100 samples from the LegalBench benchmark with the assistance of two PhD candidates with legal training as domain experts. We measure privacy risk using the average reconstruction score of the adversary model (GPT-4o) when inferring specific legal entities from obfuscated queries. The score ranges from 0 to 2, where 0 indicates no identifiable privacy leakage and 2 indicates substantial privacy leakage. Further, we measure utility validity as the percentage of obfuscated queries judged by the experts to remain legally sound and applicable after semantic abstraction, with particular attention to whether sufficient task-relevant legal information is retained for the intended legal reasoning. GS-Chaff achieves an average reconstruction risk of 0.56 out of 2 and a utility validity of 96.0%, indicating that, in most evaluated cases, semantic abstraction reduces entity-level exposure without removing sufficient legal context to make the resulting queries unsound or inapplicable. A detailed case study illustrating the semantic stealthiness of GS-Chaff and the behavior of the α-guided abstraction mechanism is provided in Appendix A.

### 4.4. Agent Architecture Comparison

We compare GS-Chaff with four alternative implementations: a single-agent two-stage implementation, a single joint-prompt implementation, a deterministic heuristic policy, and a non-LLM entity abstraction method. The single-agent variant uses the same number of SLM calls as GS-Chaff, while the joint-prompt variant predicts α, β, and the generated queries in a single prompt. Table 6 reports task accuracy, Privacy Protection Rate (PPR), token overhead, and latency.

Compared with the single-agent implementation using the same number of SLM calls, GS-Chaff improves MedQA and LegalBench accuracy by 1.22 and 1.24 percentage points, respectively, and improves PPR by 4.45 and 4.82 percentage points, with only 14 additional tokens and 0.08 s of latency per sample. GS-Chaff also achieves higher accuracy and PPR than the joint-prompt, deterministic heuristic, and non-LLM abstraction variants, while the joint-prompt and non-LLM variants have lower computational overhead. These results support the separation of privacy policy selection and semantic query generation with moderate additional cost.

***Computational Cost.*** To verify the applicability of our framework, we measure the computational cost needed for implementation. We calculate average token consumption (Avg. Token) and average Time consumption (Avg. Time) per sample to compare computational overhead against fixed baselines. The reported time measures the local processing overhead associated with GS-Chaff rather than complete end-to-end cloud inference latency. We evaluate against a fixed baseline (the standard method without additional sampling) and a fixed β=5 cases. Table 7 demonstrates that GS-Chaff achieves competitive efficiency compared to the fixed prior.

**Table 7:** Average local computational overhead per sample.

Method	Avg. Token	Avg. Time (s)
GS-Chaff (Ours)	324	2.6
Fixed (no sampling)	142	0
Fixed (β=5)	785	4.8
***SLM Backbone Comparison.*** To evaluate the influence of the local SLM backbone, we further compare Llama-3.2-1B, Qwen2.5-1.5B, Gemma-2-2B [54], and Phi-3-mini [55] under the same experimental configuration. We report task utility across four benchmarks, together with Top-1 real-query identification accuracy and local deployment overhead. The attack advantage (Adv.) is calculated relative to the random-guessing reference of 21.2%, with lower Top-1 identification accuracy and attack advantage indicating stronger privacy protection.

As shown in Table 8, GS-Chaff maintains consistent utility and privacy performance across the four evaluated SLM backbones. The differences on StrategyQA and MuSiQue are relatively small, while MedQA accuracy increases from 84.51% with Llama-3.2-1B to 86.50% with Phi-3-mini, and LegalBench accuracy increases from 81.25% to 84.45%. Meanwhile, the Top-1 real-query identification accuracy decreases from 23.85% to 21.28%, approaching the 21.2% random-guessing reference. The improved performance of larger SLMs is accompanied by higher local deployment overhead. Peak memory increases from 2.43 GB for Llama-3.2-1B to 7.81 GB for Phi-3-mini, while local inference time increases from 1.24 s to 3.76 s. Qwen2.5-1.5B and Gemma-2-2B provide intermediate trade-offs between model capability and local resource consumption, indicating that the SLM backbone can be selected according to the resource constraints of the client device.

## 5. Discussion

Although GS-Chaff demonstrates effectiveness under the single-session attack settings evaluated in this study, several stronger threat settings remain to be addressed. These include the following scenarios:

- Cross-session or long-term user profiling, where an attacker links queries from multiple sessions to construct a long-term user behavior profile;
- Broader side-channel analysis, where an attacker infers the user’s real intent from request frequency, network traffic patterns, server logs, or other metadata beyond the token-length and end-to-end latency features evaluated in this study;
- Local client compromise or real-query index leakage, where an attacker obtains the locally stored index used to retrieve the response corresponding to the real query;
- Broader adaptive semantic attacks, where attackers exploit learned semantic or representation-level cues to distinguish the real query under different domains and policy configurations.

In GS-Chaff, privacy protection is provided through the coordinated actions of two SLM-driven agents: the *privacy policy agent* adaptively determines the abstraction and chaffing parameters, while the *generative semantic chaffing agent* produces semantically consistent chaff queries to conceal the real query. This design supports privacy-preserving inference over natural-language text queries from different application domains, without requiring modification of the cloud-based LLM. In the current study, GS-Chaff is evaluated in the context of privacy-preserving inference over natural-language text queries. Extending the framework to sensor-derived inputs and multimodal settings will require additional mechanisms for semantic conversion, cross-modal alignment, and deployment on resource-constrained edge platforms, which we leave for future work.

## 6. Conclusions

This work presents GS-Chaff, a multi-agent framework for privacy-preserving LLM inference over natural-language text queries through user-intent hiding. GS-Chaff employs a privacy policy agent to adaptively determine semantic abstraction and chaffing parameters, together with a generative semantic chaffing agent that produces semantically consistent chaff queries. Experimental results on the evaluated text-based benchmarks demonstrate that the coordinated agent design reduces real-query identification risk under the evaluated attack settings while maintaining task utility on these benchmarks. By leveraging lightweight SLMs for policy control and semantic query generation, GS-Chaff provides a client-side privacy protection mechanism without requiring modifications to the cloud-based LLM. Future work will investigate system-level protections and long-term or cross-session attacks. Extending GS-Chaff to sensor-derived inputs and multimodal settings will require further exploration of semantic conversion, cross-modal alignment, and deployment on resource-constrained edge platforms.

## Figures and Tables

**Table 1:** Qualitative comparison of representative prompt-obfuscation methods.

Method	Privacy Mechanism	Computational Cost	Semantic Preservation	Adversarial Resistance	Deployment Feasibility
EmojiPrompt	Symbol-based prompt obfuscation	Low	Moderate	Inference attacks evaluated	High
Petridish	Prompt obfuscation with confidential execution	Moderate–High	High	Prompt reconstruction protection	Moderate
Lock and Decode	Obfuscated prompt encoding	Low–Moderate	Moderate	Security-bypass setting	High
WordGame	Query–response obfuscation	Low	Moderate	Jailbreak attack setting	High
CodeCipher	Learned code obfuscation	Moderate–High	High for code tasks	Source-code protection	Limited

**Table 2:** Utility evaluation of privacy-preserving inference strategies across multiple benchmarks under local and cloud-based LLM settings.

Method	Model	StrategyQA		MuSiQue			MedQA	LegalBench
		Acc.	AUC	F1	ROUGE-L	EM	Acc.	Acc.
**Local Inference**								
-	Llama2-7B	0.602	0.581	0.484	0.485	0.321	0.304	0.320
-	Vicuna-13B	0.646	0.628	0.417	0.416	0.225	0.315	0.340
**Cloud Inference**								
No Privacy Protection	GPT-3.5-Turbo	0.751	0.737	0.604	0.605	0.439	0.602	0.650
No Privacy Protection	GPT-4-Turbo	0.803	0.798	0.663	0.664	0.503	0.866	0.840
No Privacy Protection	GPT-4o	0.791	0.784	0.721	0.721	0.557	0.870	0.850
Text2Text	GPT-3.5-Turbo	0.528	0.496	0.019	0.019	0.010	0.260	0.280
Text2Text	GPT-4-Turbo	0.537	0.506	0.028	0.027	0.014	0.270	0.290
Text2Text	GPT-4o	0.533	0.500	0.021	0.020	0.012	0.275	0.295
Paraphraser	GPT-3.5-Turbo	0.489	0.478	0.076	0.076	0.028	0.280	0.300
Paraphraser	GPT-4-Turbo	0.546	0.523	0.060	0.061	0.026	0.290	0.310
Paraphraser	GPT-4o	0.537	0.515	0.072	0.072	0.038	0.295	0.315
Confusion Prompt	GPT-3.5-Turbo	0.723	0.726	0.606	0.605	0.445	0.580	0.620
Confusion Prompt	GPT-4-Turbo	0.741	0.743	0.633	0.634	0.495	0.810	0.780
Confusion Prompt	GPT-4o	0.733	0.739	0.685	0.684	0.535	0.815	0.790
**GS-Chaff (Ours)**	GPT-3.5-Turbo	**0.745**	**0.730**	**0.598**	**0.600**	**0.435**	**0.595**	**0.640**
**GS-Chaff (Ours)**	GPT-4-Turbo	**0.795**	**0.790**	**0.658**	**0.660**	**0.500**	**0.860**	**0.835**
**GS-Chaff (Ours)**	GPT-4o	**0.788**	**0.781**	**0.718**	**0.718**	**0.552**	**0.865**	**0.845**

**Table 3:** Adversarial real-query identification rate under the evaluated multi-agent consensus attack setting. ↓ indicates that lower values are better.

Method	Mechanism	Real-Query Identification Rate (%) ↓
Random Guess	Sampling-based Estimation	21.2 ± 1.5
DP Perturbation	Differential Privacy Noise	92.5 ± 2.1
Entity Replacement	Rule-based Substitution	65.0 ± 4.2
**GS-Chaff (Ours)**	**Generative Semantic Obfuscation**	**22.5 ± 1.8**

**Table 4:** Ablation study on the privacy policy components. Dynamic parameterization in GS-Chaff improves the trade-off between task accuracy (Acc.) and Privacy Protection Rate (PPR) across domain-specific benchmarks. ↑ indicates that higher values are better.

Configuration	Metric	MedQA	LegalBench
Full GS-Chaff (Qwen2.5-1.5B, dynamic α,β)	Acc.	86.32 ± 1.05	83.74 ± 1.33
	PPR ↑	92.85 ± 0.72	91.02 ± 0.95
Full GS-Chaff (Llama-3.2-1B, dynamic α,β)	Acc.	84.51 ± 1.24	81.25 ± 1.56
	PPR ↑	91.20 ± 0.88	89.53 ± 1.04
Static β=5	Acc.	82.15 ± 1.52	79.48 ± 1.82
	PPR ↑	88.47 ± 1.15	86.41 ± 1.28
Static α=3	Acc.	81.38 ± 1.45	78.12 ± 1.67
	PPR ↑	87.92 ± 1.30	85.25 ± 1.42
Static α=3,β=5	Acc.	76.85 ± 2.10	72.50 ± 2.55
	PPR ↑	80.54 ± 1.95	78.10 ± 2.21
w/o α	Acc.	73.10 ± 1.85	69.80 ± 2.10
	PPR ↑	79.25 ± 1.75	76.50 ± 2.00
w/o β	Acc.	84.10 ± 1.05	80.95 ± 1.35
	PPR ↑	61.50 ± 2.40	59.80 ± 2.55

**Table 5:** Robustness to a limited side-channel attack based on token length and end-to-end request latency. ↓ indicates that lower values are better.

Signal	Top-1 acc (%) ↓	MRR ↓
Random guess ($\frac{1}{\beta }$)	21.2 ± 1.5	0.372 ± 0.014
Per-query token length	23.1 ± 1.6	0.386 ± 0.015
End-to-end latency	23.8 ± 1.7	0.392 ± 0.016
Token length + latency	24.6 ± 1.8	0.401 ± 0.017

**Table 6:** Comparison of agent architectures and alternative implementations. ↓ indicates that lower values are better and ↑ indicates that higher values are better.

Method	SLM Calls	MedQA Acc. ↑	LegalBench Acc. ↑	MedQA PPR ↑	LegalBench PPR ↑	Avg. Tokens ↓	Avg. Time (s) ↓
**GS-Chaff**	2	**86.32 ± 1.05**	**83.74 ± 1.33**	**92.85 ± 0.72**	**91.02 ± 0.95**	324	2.60
Single-Agent Implementation	2	85.10 ± 1.18	82.50 ± 1.40	88.40 ± 1.10	86.20 ± 1.25	310	2.52
Single Joint-Prompt	1	82.40 ± 1.45	79.80 ± 1.62	83.10 ± 1.35	81.50 ± 1.50	215	1.65
Deterministic Heuristic Policy	1	81.20 ± 1.50	78.50 ± 1.68	80.50 ± 1.60	78.10 ± 1.75	260	2.10
Non-LLM Entity Abstraction	0	76.50 ± 1.90	73.20 ± 2.10	71.20 ± 2.15	68.40 ± 2.30	180	1.35

**Table 8:** Comparison of SLM backbones in GS-Chaff. ↓ indicates that lower values are better and ↑ indicates that higher values are better.

SLM Backbone	StrategyQA		MuSiQue			MedQA	LegalBench	Privacy		Local Overhead	
	Acc. ↑	AUC ↑	F1 ↑	ROUGE-L ↑	EM ↑	Acc. ↑	Acc. ↑	Top-1 ID (%) ↓	Adv. (pp) ↓	Memory ↓	Time ↓
Llama-3.2-1B	78.82 ± 1.15	78.14 ± 1.10	71.23 ± 1.25	71.21 ± 1.20	54.62 ± 1.35	84.51 ± 1.24	81.25 ± 1.56	23.85 ± 1.18	2.65 ± 0.32	2.43 GB	1.24 s
Qwen2.5-1.5B	79.15 ± 1.02	78.43 ± 0.98	71.58 ± 1.12	71.56 ± 1.08	55.01 ± 1.21	86.32 ± 1.05	83.74 ± 1.33	22.54 ± 1.05	1.34 ± 0.22	3.18 GB	1.58 s
Gemma-2-2B	79.31 ± 0.91	78.62 ± 0.86	71.72 ± 0.95	71.70 ± 0.91	55.15 ± 1.04	86.45 ± 0.98	84.12 ± 1.22	21.62 ± 0.92	0.42 ± 0.15	4.26 GB	2.12 s
Phi-3-mini	79.40 ± 0.83	78.71 ± 0.79	71.80 ± 0.88	71.78 ± 0.84	55.20 ± 0.96	86.50 ± 0.85	84.45 ± 1.05	21.28 ± 0.81	0.08 ± 0.08	7.81 GB	3.76 s

## Data Availability

The StrategyQA dataset utilized in this study is openly accessible at https://github.com/eladsegal/strategyqa, accessed on 1 May 2026; The MuSiQue dataset can be found at https://github.com/stonybrooknlp/musique, accessed on 23 April 2026; The MedQA dataset is available for free at https://github.com/jind11/MedQA, accessed on 11 May 2026; The LegalBench dataset is available at https://github.com/HazyResearch/legalbench, accessed on 9 May 2026.

## Abbreviations

The following abbreviations are used in this manuscript:

LLM	Large language model
GS-Chaff	Generative Semantic Chaffing
LDP	Local differential privacy
DP	Differential privacy
QA	Question answering
NLP	Natural language processing
AUC	Area under the receiver operating characteristic curve
ROC	Receiver operating characteristic
F1	F1 score
ROUGE-L	Recall-Oriented Understudy for Gisting Evaluation–Longest Common Subsequence
EM	Exact match
GPT	Generative Pre-trained Transformer
GPT-4o	Generative Pre-trained Transformer 4 Omni

## Appendix A. Case Study on Semantic Stealthiness

In this appendix, we illustrate how GS-Chaff achieves intent obfuscation at the instance level through a legal-domain query inspired by the LegalBench style. The focus is on *mechanism interpretability*: showing the privacy parameters 〈α,β〉 output by the policy agent and how the generated chaff queries satisfy the semantic abstraction coverage required by α. This case study is intended only to illustrate the behavior of the α-guided abstraction mechanism and does not constitute a formal verification of candidate-set coverage.

### Appendix A.1. Real Query and Policy Output

The real user query (Real Query) is:

“What personal legal liabilities may corporate executives face in securities fraud cases in the US?”

The policy agent outputs: 〈α=4,β=5〉

Explanation: This query belongs to the legal domain and involves potentially high-sensitivity decision consequences (liability types, risk assessment). Therefore, the policy favors a moderate obfuscation strength (β relatively high to increase uncertainty) while maintaining medium-level abstraction (α avoids excessive generalization that may distort reasoning). Configurations around α≈ 3–4 and β≈5 align with the empirical distributions reported in Figure 3.

### Appendix A.2. Meaning of α = 4

Here, α represents the minimum number of distinct but semantically reasonable candidate entities that the abstracted description should cover. For this case, we abstract the real query into a description without specific case details but retaining core legal semantics:

“Individual legal liability/consequences of corporate executives in securities-related illegal situations”

Within this abstraction, at least the following 4 semantic scenarios satisfy α=4:

1. Securities fraud
2. Insider trading
3. Market manipulation
4. Disclosure violations

Additional possibilities include accounting fraud, breach of fiduciary duty, or regulatory enforcement consequences. Thus, α=4 ensures that subsequent chaff queries fall within the common semantic shell of securities law liability/corporate executive liability, maintaining compatibility with at least 4 specific scenarios without revealing the unique real intent.

### Appendix A.3. Generated Query Set (β = 5)

With β=5, GS-Chaff generates 5 queries: 1 real query + 4 chaff queries. Example (randomly permuted):

- Q1: *In securities-related litigation, what personal liabilities do corporate directors and executives typically need to assume?*
- Q2 (Real): **What personal legal liabilities may corporate executives face in securities fraud cases in the US?**
- Q3: *What legal consequences may company management face when violating securities regulatory regulations?*
- Q4: *Under the US legal system, what obligations and responsibilities might managers have towards investors?*
- Q5: *What types of liability investigations are generally triggered by fraudulent or misleading conduct in the securities market?*

### Appendix A.4. Why the Chaff Queries Satisfy α = 4

Each chaff query maintains the same abstract semantic shell and does not narrow the candidate entity set below 4:

- Q1: Directors/executives in securities litigation–compatible with fraud, insider trading, disclosure violations, market manipulation → α=4
- Q3: Violations of securities regulations–broad enough to cover multiple legal scenarios → α=4
- Q4: Managerial obligations towards investors–spans multiple investor-related scenarios → α=4
- Q5: Fraud/misleading conduct–general category covering multiple cases → α=4

All chaff queries share the same high-level semantic theme (securities law/corporate executive liability) and role (executives/managers) while avoiding unique details that would reduce coverage.

### Appendix A.5. Implications for Semantic Stealthiness

In this example, an attacker observes a set of semantically consistent queries rather than a single standout real query plus unrelated noise. The real query is naturally embedded, increasing uncertainty in identifying the true intent. This aligns with the real-query identification rate reduction observed in Table 3 of the main text at the mechanism level.

## References

[1] Brown T., Mann B., Ryder N., Subbiah M., Kaplan J.D., Dhariwal P., Neelakantan A., Shyam P., Sastry G., Askell A. (2005). Language models are few-shot learners. arXiv.

[2] Bommasani R. (2021). On the opportunities and risks of foundation models. arXiv.

[3] Weidinger L., Mellor J., Rauh M., Griffin C., Uesato J., Huang P.S., Cheng M., Glaese M., Balle B., Kasirzadeh A. (2021). Ethical and social risks of harm from language models. arXiv.

[4] Wan Z., Guo C., Hu B., Du J., Mou X., Zhang J. (2025). LLM-Based V2X Multi-Model Sensor Data Fusion for Improved Road Safety and Data Privacy. Proceedings of the 2025 34th International Conference on Computer Communications and Networks (ICCCN).

[5] Bender E.M., Gebru T., McMillan-Major A., Shmitchell S. On the dangers of stochastic parrots: Can language models be too big?. Proceedings of the 2021 ACM Conference on Fairness, Accountability, and Transparency.

[6] Carlini N., Tramer F., Wallace E., Jagielski M., Herbert-Voss A., Lee K., Roberts A., Brown T., Song D., Erlingsson U. Extracting training data from large language models. Proceedings of the 30th USENIX security symposium (USENIX Security 21).

[7] Li H., Chen Y., Luo J., Wang J., Peng H., Kang Y., Zhang X., Hu Q., Chan C., Xu Z. (2023). Privacy in large language models: Attacks, defenses and future directions. arXiv.

[8] Alsboui T.A., Al-Aqrabi H., Hill R., Iram S. An Approach to Privacy-Preserving Distributed Intelligence for the Internet of Things. Proceedings of the IoTBDS.

[9] de Castro L., Polychroniadou A., Escudero D. Privacy-preserving large language model inference via GPU-accelerated fully homomorphic encryption. Proceedings of the Neurips Safe Generative AI Workshop 2024.

[10] Jesutosin A.O., Akpan I.E. (2025). Encrypted Prompting and Secure Inference in LLM-as-a-Service Models: A Differential Privacy and Trusted Execution Environment Framework. https://www.researchgate.net/publication/399040924_Encrypted_Prompting_and_Secure_Inference_in_LLM-as-a-Service_Models_A_Differential_Privacy_and_Trusted_Execution_Environment_Framework.

[11] Kalodanis K., Papadopoulos S., Feretzakis G., Rizomiliotis P., Anagnostopoulos D. (2025). SecureLLM: A Unified Framework for Privacy-Focused Large Language Models. Appl. Sci..

[12] Cui Y., Cao X., Zhu G., Nie J., Xu J. (2025). Edge perception: Intelligent wireless sensing at network edge. IEEE Commun. Mag..

[13] Li X., Yin Z., Gu X., Shen B. (2025). Anti-adversarial Learning: Desensitizing Prompts for Large Language Models. arXiv.

[14] Yang T., Zhu X., Gurevych I. (2025). Robust utility-preserving text anonymization based on large language models. Proceedings of the 63rd Annual Meeting of the Association for Computational Linguistics (Volume 1: Long Papers).

[15] Jia F., Fonsati A., Gudmundsson K. (2024). Natural language communication with sensor data through a LLM-integrated protocol: A case study. Proceedings of the International Conference on Computing in Civil and Building Engineering.

[16] Osamy W., Khedr A.M., Salim A., Al Ali A.I., El-Sawy A.A. (2022). Coverage, deployment and localization challenges in wireless sensor networks based on artificial intelligence techniques: A review. IEEE Access.

[17] Abou Ali M., Dornaika F., Charafeddine J. (2025). Agentic AI: A comprehensive survey of architectures, applications, and future directions. Artif. Intell. Rev..

[18] Cheng Y., Ye H., Li H.H., Sun J., Chen Y. (2026). PrivAct: Internalizing Contextual Privacy Preservation via Multi-Agent Preference Training. arXiv.

[19] Xu N., Gong Z., Ran R., Tang J., Wen W., Ding C. (2025). FHE-Agent: Automating CKKS Configuration for Practical Encrypted Inference via an LLM-Guided Agentic Framework. arXiv.

[20] Rivest R.L. (1998). Chaffing and winnowing: Confidentiality without encryption. CryptoBytes (RSA Lab.).

[21] Cheng Q., Shi Z., Yuan W., Ma Y., Wang J., Sun G. (2026). LLM-Enabled LAWNs: Toward Integrated Sensing, Communication, and Control. IEEE Netw..

[22] Yao A.C.C. (1986). How to generate and exchange secrets. Proceedings of the 27th Annual Symposium on Foundations of Computer Science (Sfcs 1986).

[23] Gentry C. Fully homomorphic encryption using ideal lattices. Proceedings of the Forty-First Annual ACM Symposium on Theory of Computing.

[24] Dwork C., McSherry F., Nissim K., Smith A. (2006). Calibrating noise to sensitivity in private data analysis. Proceedings of the Theory of Cryptography Conference.

[25] Yuan J., Xu L., Jiang H., Rahmani H., Soh D.W., Liu J. (2022). PrivateChat: A Secure Encrypted Communication Framework with Black-Box LLMs. https://openreview.net/forum?id=SX2Z5tgiUu.

[26] Xue Y., Liu L., Luo Y., Sun B., Fu S. (2025). FLUTE: FSS-Based Secure Two-Party LLM Inference Using Partial Transformer Encryption. IEEE Trans. Dependable Secur. Comput..

[27] Wang B., Garcia L.A., Srivastava M. (2024). PrivacyOracle: Configuring sensor privacy firewalls with large language models in smart built environments. Proceedings of the 2024 IEEE Security and Privacy Workshops (SPW).

[28] Bae Y., Kim M., Lee J., Kim S., Kim J., Choi Y., Mireshghallah N. (2025). Privacy-Preserving LLM Interaction with Socratic Chain-of-Thought Reasoning and Homomorphically Encrypted Vector Databases. arXiv.

[29] Zimerman I., Adir A., Aharoni E., Avitan M., Baruch M., Drucker N., Lerner J., Masalha R., Meiri R., Soceanu O. (2024). Power-softmax: Towards secure llm inference over encrypted data. arXiv.

[30] Kumar M., Xue J., Zheng M., Lou Q. (2025). Tfhe-coder: Evaluating llm-agentic fully homomorphic encryption code generation. arXiv.

[31] Tan Y., Tan C., Mi Z., Chen H. Pipellm: Fast and confidential large language model services with speculative pipelined encryption. Proceedings of the 30th ACM International Conference on Architectural Support for Programming Languages and Operating Systems.

[32] Zhang J., Tian Z., Zhu M., Song Y., Sheng T., Yang S., Du Q., Liu X., Huang M., Li D. DYNTEXT: Semantic-aware dynamic text sanitization for privacy-preserving LLM inference. Proceedings of the Findings of the Association for Computational Linguistics: ACL 2025.

[33] Albanese F., Ciolek D., D’Ippolito N. (2023). Text sanitization beyond specific domains: Zero-shot redaction & substitution with large language models. arXiv.

[34] Fu W., Wang H., Gao J., Wan G., Jiang T. (2025). Sanitize Your Responses: Mitigating Privacy Leakage in Large Language Models. arXiv.

[35] Thareja R., Gupta G., Nakov P., Vepakomma P., Lukas N. Sanitizing Medical Documents with Differential Privacy using Large Language Models. Proceedings of the Second Workshop on GenAI for Health: Potential, Trust, and Policy Compliance.

[36] Tong M., Chen K., Yuan X., Liu J., Zhang W., Yu N., Zhang J. (2025). On the vulnerability of text sanitization. Proceedings of the 2025 Conference of the Nations of the Americas Chapter of the Association for Computational Linguistics: Human Language Technologies (Volume 1: Long Papers).

[37] Carpentier R., Zhao B.Z.H., Asghar H.J., Kaafar D. (2024). Preempting Text Sanitization Utility in Resource-Constrained Privacy-Preserving LLM Interactions. arXiv.

[38] Meisenbacher S., Klymenko A., Bodea A.E., Matthes F. The Double-edged Sword of LLM-based Data Reconstruction: Understanding and Mitigating Contextual Vulnerability in Word-level Differential Privacy Text Sanitization. Proceedings of the 24th Workshop on Privacy in the Electronic Society—WPES ’25.

[39] Lin S., Hua W., Wang Z., Jin M., Fan L., Zhang Y. (2025). Emojiprompt: Generative prompt obfuscation for privacy-preserving communication with cloud-based llms. Proceedings of the 2025 Conference of the Nations of the Americas Chapter of the Association for Computational Linguistics: Human Language Technologies (Volume 1: Long Papers).

[40] Gim I., Li C., Zhong L. (2024). Confidential prompting: Protecting user prompts from cloud llm providers. arXiv.

[41] Yadavalli S., Shivaswaroopa A., Raval M., Bhowmik A., Shaikh A., Arakeri M. Lock and Decode: Obfuscated Prompt Cryptography as a Mechanism for Circumventing Large Language Model Security Paradigms. https://ssrn.com/abstract=5128143.

[42] Zhang T., Cao B., Cao Y., Lin L., Mitra P., Chen J. (2025). Wordgame: Efficient & effective llm jailbreak via simultaneous obfuscation in query and response. Proceedings of the Findings of the Association for Computational Linguistics: NAACL 2025.

[43] Lin Y., Wan C., Fang Y., Gu X. (2024). CodeCipher: Learning to Obfuscate Source Code Against LLMs. arXiv.

[44] Yang H., Zhao R., Wang G., Deng Z. (2025). GAMA: A General Anonymizing Multi-Agent System for Privacy Preservation Enhanced by Domain Rules and Disproof Mechanism. arXiv.

[45] Chen J., Yang J., Zeng Z., Huang Z., Li J., Wang Y. SecureGov-Agent: A Governance-Centric Multi-Agent Framework for Privacy-Preserving and Attack-Resilient LLM Agents. Proceedings of the ICCSMT ’25: Proceedings of the 2025 6th International Conference on Computer Science and Management Technology.

[46] Gosmar D., Dahl D.A. (2025). Sentinel agents for secure and trustworthy agentic ai in multi-agent systems. arXiv.

[47] Li J., Tang T., Zhao W.X., Nie J.Y., Wen J.R. (2024). Pre-trained language models for text generation: A survey. ACM Comput. Surv..

[48] Androutsopoulos I., Malakasiotis P. (2010). A survey of paraphrasing and textual entailment methods. J. Artif. Intell. Res..

[49] Mai P., Yang Y., Yan R., Ye R., Pang Y. (2023). ConfusionPrompt: Practical private inference for online large language models. arXiv.

[50] Geva M., Khashabi D., Segal E., Khot T., Roth D., Berant J. (2021). Did aristotle use a laptop? A question answering benchmark with implicit reasoning strategies. Trans. Assoc. Comput. Linguist..

[51] Trivedi H., Balasubramanian N., Khot T., Sabharwal A. (2022). MuSiQue: Multihop Questions via Single-hop Question Composition. Trans. Assoc. Comput. Linguist..

[52] Jin D., Pan E., Oufattole N., Weng W.H., Fang H., Szolovits P. (2021). What disease does this patient have? a large-scale open domain question answering dataset from medical exams. Appl. Sci..

[53] Guha N., Nyarko J., Ho D., Ré C., Chilton A., Chohlas-Wood A., Peters A., Waldon B., Rockmore D., Zambrano D. (2023). Legalbench: A collaboratively built benchmark for measuring legal reasoning in large language models. Adv. Neural Inf. Process. Syst..

[54] Team G., Riviere M., Pathak S., Sessa P.G., Hardin C., Bhupatiraju S., Hussenot L., Mesnard T., Shahriari B., Ramé A. (2024). Gemma 2: Improving open language models at a practical size. arXiv.

[55] Abdin M., Aneja J., Awadalla H., Awadallah A., Awan A.A., Bach N., Bahree A., Bakhtiari A., Bao J., Behl H. (2024). Phi-3 Technical Report: A Highly Capable Language Model Locally on Your Phone. arXiv.

